# Recent trends in population levels and correlates of occupational and leisure sitting time in full-time employed Australian adults

**DOI:** 10.1371/journal.pone.0195177

**Published:** 2018-04-12

**Authors:** Anne Loyen, Tien Chey, Lina Engelen, Adrian Bauman, Jeroen Lakerveld, Hidde P. van der Ploeg, Johannes Brug, Josephine Y. Chau

**Affiliations:** 1 Department of Public and Occupational Health, Amsterdam Public Health research institute, VU University Medical Center, Amsterdam, the Netherlands; 2 Prevention Research Collaboration, Sydney School of Public Health and Charles Perkins Centre, the University of Sydney, Sydney, Australia; 3 Department of Epidemiology and Biostatistics, Amsterdam Public Health research institute, VU University Medical Center, Amsterdam, the Netherlands; 4 Amsterdam School for Communication Research, University of Amsterdam, Amsterdam, the Netherlands; Medical University of Vienna, AUSTRIA

## Abstract

This study aimed to explore the trend in population levels, as well as the correlates, of occupational and leisure sitting time in full-time employed Australian adults between 2007 and 2015. We used data from the 2007/08, 2011/12 and 2014/15 Australian Health Surveys, in which nationally representative samples of the Australian population were interviewed. Full-time (≥35 hours/week) employed respondents reported sitting time at work and during leisure on a usual workday. Trends over time and associations between socio-demographic and health-related characteristics and sitting time were analysed in the combined dataset using multivariable logistic regression models. Over 21,000 observations were included in the analyses. Across the three surveys, approximately 51% of the respondents reported ≥4 hours/workday occupational sitting time, 40% reported ≥4 hours/workday leisure sitting time, and 55% reported ≥7 hours/workday combined occupational and leisure sitting time. There were no clear trends over time. All potential correlates were associated with occupational sitting time and all but educational level were associated with leisure sitting time. The directions of the associations with gender, age and leisure-time physical activity were reversed for occupational sitting time and leisure sitting time. These findings show that the average levels of occupational and leisure sitting time on workdays were high but stable over the past decade. The observed differences in correlates of occupational and leisure sitting time demonstrate the need to assess and address sedentary behaviour domains separately in research and policy.

## Background

Sedentary behaviour is increasingly recognised as an important health risk. It is defined as any waking low-energy behaviour in a sitting, reclining or lying position.[1] The time spent in sedentary behaviours has been associated with increased morbidity and mortality.[2] The risk of all-cause mortality is shown to increase in adults who sit more than seven to eight hours/day,[3] but these associations seem to be attenuated by physical activity levels,[2–4] and even eliminated by very high levels of moderate to vigorous physical activity.[4] For health purposes it thus seems important to limit the time spent in sedentary behaviours, in addition to being sufficiently physically active. This is also reflected in multiple national physical activity guidelines, which recommend to minimise and break up sitting time.[5–7]

Surveillance data of sedentary behaviour can be used to assess population levels, identify groups with high levels of sedentary time, and inform public health strategies. Comparable trend data are especially interesting since they allow monitoring change over time, but these data are scarce and estimates vary. For example, a repeated cross-sectional study in the Netherlands showed that non-occupational sitting time, and especially screen time, increased between 1975 and 2005.[8] However, a longitudinal 10-year follow-up study in Canada showed stability in total sitting time since 1995/1997,[9] whereas a repeated cross-sectional study based on Australian Time Use Surveys showed a slight but significant increase in non-occupational sedentary activities in the same period.[10] And while a repeated cross-sectional European study reported a decrease in high sitting time (defined as ≥7.5 hours/day) between 2002 and 2013,[11] a repeated cross-sectional Danish study showed (small) increases in both leisure and occupational sitting time between 2007 and 2010.[12]

A promising source of recent trend data of sedentary time in Australian adults is the Australian Health Survey. The Australian Health Survey is a series of interview-based health surveys conducted regularly since 1989 in representative samples of the Australian national population.[13] In the three most recent surveys, conducted in 2007/08, 2011/12 and 2014/15, full-time employed participants were asked to report sedentary behaviour during work and leisure. Using these data, this study aims to explore the trend, as well as the socio-demographic and health-related correlates, of occupational and leisure sitting time in full-time employed adults across the 2007/08, 2011/12 and 2014/15 Australian Health Surveys.

## Materials and methods

### Subjects and sampling

The Australian Bureau of Statistics (ABS) Australian Health Survey consist of nationally representative samples of the Australian population from randomly selected private dwellings in urban and rural areas of all states and territories.[14–16] In each household, one adult (≥18 years) and -where applicable- one child (0–17 years) was randomly selected. In this study, we only used data from respondents aged ≥15 years reporting to work ≥35 hours/week, as these respondents provided information on both their occupational as well as their leisure sitting time (see details below). Trained interviewers conducted personal interviews using a computer-assisted instrument. Survey response rates for fully and adequately responding households in 2007/08, 2011/12 and 2014/15 were 90.6%, 84.8% and 82.0%, respectively.[14–16] Respondents provided informed consent to the ABS at the time of the interview and permission to access the survey data was granted by the ABS to authors TC and JYC.

### Sedentary behaviour measures

The Australian Health Survey measured sedentary behaviour in 2007/08, 2011/12 and 2014/15 using the same questions. Full-time employed (≥35 hours/week) respondents reported the time spent sitting at work on a usual workday, and all respondents reported the time spent sitting for leisure (including screen time) on a usual work or week day. In the current analyses, only the full-time employed respondents were included, as they provided information on both occupational and leisure sitting time. Occupational sitting time and leisure sitting time were dichotomised into sitting less than 4 hours/workday or 4 hours/workday or more, based on the median of these variables. In addition, we summed respondents’ self-reported occupational and leisure sitting time to create a combined occupational and leisure sitting time variable. This variable was included to enable comparisons to previous studies that focused on total sedentary time, as occupational and leisure sedentary time comprise the largest part of total sedentary time, especially in full-time employed adults. Respondents who reported sitting ≥7 hours/workday for work and leisure combined were categorised as having high sitting time, based on a meta-analysis showing increased risk of all-cause mortality around seven to eight hours/day of sedentary time.[3]

### Other measures

Respondents provided information about their gender, age (categorised into 15–34 years, 35–54 years and >55 years old), educational level (‘no university’ and ‘university’), household income (in tertiles and ‘not stated’), and self-rated health (dichotomised into poor, fair and good; and very good and excellent). Moreover, respondents reported the time they spent in leisure-time physical activities, based on the frequency and duration of walking, moderate, and vigorous intensity physical activities in the last week. Respondents who reported ≥150 minutes/week physical activity were defined as ‘sufficiently active’.[17] respondents who reported 30–149 minutes/week were defined as ‘insufficiently active’ and those who reported <30 minutes/week were defined as ‘inactive’.[18] In addition, the interviewers used digital scales to measure respondents’ weight and a stadiometer to measure their height. Body mass index (BMI) was calculated as kg/m^2^ and categorised into underweight (<18.5), normal weight (18.5–<25.0), overweight (25.0–<30.0) and obese (≥30.0).[19] The 2007/08 and 2011/12 surveys contain missing BMI data, whereas there was no missing weight status data in the 2014/15 survey.

### Statistical analyses

We analysed the data using SAS software, version 9.3 (SAS Institute Inc., Cary NC, USA). The three (complete) survey samples were weighted by weights provided by the ABS to reflect the population demographics at the time of survey and to account for probability of being sampled and differential response rates across the population. Details about this process are described elsewhere.[14–16] In addition, the survey samples were gender and age standardised to the 2011/12 survey. Data from the surveys were combined in one dataset with an indicator variable for the year of survey (2007/08, 2011/12 and 2014/15) and a continuous ‘year’ variable (with the values 1 (2007/08), 5 (2011/12) and 8 (2014/15)) for linear trend analyses. All analyses were conducted in a subsample consisting of full-time employed adults. We ran multivariable logistic regression analyses, adjusted for all abovementioned socio-demographic and health-related variables, to assess the trend in ≥4 hours/workday occupational sitting time, ≥4 hours/workday leisure sitting time and ≥7 hours/workday combined occupational and leisure sitting time across the survey categories (reference: 2007/08) and linear associations per year. In addition, we ran multivariable logistic regression analyses, adjusted for all (other) socio-demographic and health-related variables, to examine the associations between potential socio-demographic and health-related correlates and ≥4 hours/workday occupational sitting time, ≥4 hours/workday leisure sitting time and ≥7 hours/workday combined occupational and leisure sitting time.

## Results

A total of 20,788 respondents were included in the 2007/08 Australian Health Survey, 20,426 in the 2011/12 survey and 19,257 in the 2014/15 survey. The normalised weighted number of respondents aged ≥15 years, reporting to work ≥35 hours/week, and who provided sitting time data was 7324, 7283 and 6670, respectively. All sample characteristics are shown in Table 1.

**Table 1:** Sample characteristics of the 2007/08, 2011/12 and 2014/15 survey samples. The study sample included Australian respondents aged ≥15 years reporting to work ≥35 hours/week. The (complete) survey samples were weighted by weights provided by the Australian Bureau of Statistics to reflect the population demographics at the time of survey and to account for probability of being sampled and differential response rates across the population, and gender and age standardised to the 2011/12 survey.

	2007/08		2011/12		2014/15	
	N	%	N	%	N	%
**Overall**	7324	100%	7283	100%	6670	100%
**Gender**						
Female	2527	34.5%	2469	33.9%	2323	34.8%
Male	4797	65.5%	4814	66.1%	4347	65.2%
**Age**						
15–34 years	2752	37.6%	2666	36.6%	2506	37.6%
35–54 years	3521	48.1%	3422	47.0%	3136	47.0%
>55 years	1051	14.4%	1195	16.4%	1028	15.4%
**Educational level**						
No university	5377	73.4%	5032	69.1%	4378	65.6%
University	1947	26.6%	2251	30.9%	2293	34.4%
**Household income**						
Lowest tertile	438	6.0%	313	4.3%	311	4.7%
Middle tertile	2758	37.7%	2497	34.3%	2181	32.7%
Highest tertile	3164	43.2%	3047	41.8%	2767	41.5%
Not stated	964	13.2%	1426	19.6%	1411	21.2%
**Self-rated health**						
Poor, fair, good	2746	37.5%	2797	38.4%	2487	37.3%
Very good, excellent	4578	62.5%	4486	61.6%	4184	62.7%
**Weight status**						
Underweight	72	1.4%	36	0.6%	83	1.2%
Normal weight	1796	34.9%	2039	33.3%	2204	33.0%
Overweight	2015	39.2%	2344	38.3%	2556	38.3%
Obese	1262	24.5%	1705	27.8%	1827	27.4%
Missing†	2179		1159		0	
**Leisure-time physical activity**						
<30 minutes/week	2838	38.8%	2495	34.3%	2091	31.4%
30–149 minutes/week	1703	23.2%	1556	21.4%	1415	21.2%
≥150 minutes/week	2783	38.0%	3232	44.4%	3164	47.4%

†The 2014/15 survey did not contain missing weight status data. To increase comparability across surveys, the percentages shown do not include the missing data.

Mean occupational sitting time (SD) on a usual workday was 227 (174) minutes/day in 2007/08, 233 (192) minutes/day in 2011/12 and 228 (192) minutes/day in 2014/15. The percentage of respondents with ≥4 hours/workday occupational sitting was 50.1%, 52.2% and 51.3%, respectively (Table 2). Compared to 2007/08, the odds ratio (OR) of ≥4 hours/workday of occupational sitting did not significantly differ in 2011/12 or 2014/15. In addition, the linear trend per year was not statistically significant.

**Table 2:** The population levels (%) and the trend (OR (95% CI) of ≥4 hours/day occupational sitting, ≥4 hours/day leisure sitting and ≥7 hours/day combined occupational and leisure sitting across the three surveys.

	≥ 4 hours/workday occupational sitting (N = 21,235)		≥ 4 hours/workday leisure sitting (N = 21,253)		≥ 7 hours/workday combined occupational and leisure sitting (N = 21,276)	
	%	OR (95% CI)*	%	OR (95% CI)*	%	OR (95% CI)*
**2007/08 (ref)**	50.1%	1.00	41.9%	1.00	54.3%	1.00
**2011/12**	52.2%	1.03 (0.96–1.10)	36.4%	**0.78 (0.73–0.84)**	53.9%	**0.92 (0.86–0.98)**
**2014/15**	51.3%	0.95 (0.88–1.02)	43.1%	1.03 (0.96–1.11)	56.2%	0.98 (0.91–1.06)
**Trend per year**	N/A	0.99 (0.98–1.00)	N/A	1.00 (0.99–1.02)	N/A	1.00 (0.98–1.01)

OR odds ratio; CI confidence interval; ref reference; N/A not applicable

Numbers in bold represent *p* <0.05

*Adjusted for gender, age, educational level, household income, self-rated health, weight status and leisure-time physical activity

For leisure sitting time on a usual workday, the mean (SD) was 205 (102) minutes/day in 2007/08, 187 (108) minutes/day in 2011/12 and 206 (132) minutes/day in 2014/15. In those years, the percentage of respondents with ≥4 hours/workday leisure sitting time was 41.9%, 36.4% and 43.1%, respectively (Table 2). Compared to 2007/08, the OR of ≥4 hours/workday of leisure sitting was significantly lower in 2011/12 but there was no difference in 2014/15. In addition, the linear trend per year was not significant.

Finally, mean combined occupational and leisure sitting time (SD) on a usual workday was 431 (204) minutes/day in 2007/08, 419 (216) minutes/day in 2011/12 and 434 (228) minutes/day in 2014/15. In addition, the percentage of respondents with ≥7 hours/workday sitting time was 54.3%, 53.9% and 56.2%, respectively (Table 2). Compared to 2007/08, the OR of ≥7 hours/workday sitting did significantly differ in 2011/12 but not in 2014/15. Again, the linear trend per year was not statistically significant.

The associations between potential correlates and sitting time are shown in Table 3. All potential correlates were associated with ≥4 hours/day occupational sitting time. The strongest associations were found for household income and educational level; people with higher household incomes and higher educational levels showed higher ORs (3.0 and 2.7, respectively) of occupational sitting ≥4 hours/day. Although less pronounced, all potential correlates were also associated with ≥4 hours/day leisure sitting time, with the exception of educational level. The associations between the potential correlates and combined occupational and leisure sitting time were similar to the associations with occupational sitting time. The directions of the associations with gender, age, and leisure-time physical activity were reversed for occupational sitting time and leisure sitting time.

**Table 3:** The associations (OR (95% CI)) of potential socio-demographic and health-related correlates with ≥4 hours/day occupational sitting, ≥4 hours/day leisure sitting, and ≥7 hours/day combined occupational and leisure sitting, in the study sample combining all three surveys.

	≥ 4 hours/workday occupational sitting (N = 21,235)	≥ 4 hours/workday leisure sitting (N = 21,253)	≥ 7 hours/workday combined occupational and leisure sitting (N = 21,276)
	OR (95% CI)*	OR (95% CI)*	OR (95% CI)*
**Gender**			
Female (*ref*)	1.00	1.00	1.00
Male	**0.71 (0.67–0.76)**	**1.34 (1.26–1.42)**	**0.83 (0.78–0.88)**
**Age**			
15–34 years (*ref*)	1.00	1.00	1.00
35–54 years	**1.50 (1.40–1.60)**	**0.80 (0.75–0.85)**	**1.27 (1.19–1.35)**
>55 years	**1.37 (1.25–1.49)**	**0.88 (0.80–0.95)**	**1.15 (1.06–1.26)**
**Educational level**			
No university (*ref*)	1.00	1.00	1.00
University	**2.71 (2.53–2.89)**	0.95 (0.89–1.01)	**2.55 (2.38–2.72)**
**Household income**			
Lowest tertile (*ref*)	1.00	1.00	1.00
Middle tertile	**1.60 (1.39–1.85)**	**1.19 (1.04–1.35)**	**1.52 (1.32–1.74)**
Highest tertile	**3.04 (2.63–3.50)**	1.11 (0.97–1.27)	**2.68 (2.34–3.08)**
Not stated	**1.95 (1.68–2.27)**	1.07 (0.93–1.23)	**1.74 (1.50–2.01)**
**Self-rated health**			
Very good, excellent (*ref*)	1.00	1.00	1.00
Poor, fair, good	**1.15 (1.08–1.22)**	**1.26 (1.19–1.34)**	**1.25 (1.17–1.33)**
**Weight status**			
Underweight	1.01 (0.74–1.37)	1.10 (0.82–1.47)	1.06 (0.78–1.44)
Normal weight (*ref*)	1.00	1.00	1.00
Overweight	**1.17 (1.08–1.26)**	1.06 (0.99–1.14)	**1.21 (1.12–1.30)**
Obese	**1.25 (1.15–1.35)**	**1.24 (1.15–1.35)**	**1.35 (1.24–1.46)**
Missing†	**1.20 (1.09–1.32)**	0.96 (0.87–1.05)	**1.20 (1.09–1.32)**
**Leisure-time physical activity**			
<30 minutes/week	**0.67 (0.62–0.71)**	**1.20 (1.12–1.28)**	**0.76 (0.71–0.81)**
30–149 minutes/week	0.93 (0.86–1.00)	1.06 (0.98–1.14)	0.95 (0.88–1.03)
≥150 minutes/week (*ref*)	1.00	1.00	1.00

OR odds ratio; CI confidence interval; ref reference

Numbers in bold represent *p* <0.05

† The 2014/15 survey did not contain missing weight status data. Therefore, these numbers are based on the 2007/08 and 2011/12 survey only.

*Adjusted for gender, age, educational level, household income, self-rated health, weight status and leisure-time physical activity

## Discussion

The aim of this study was to explore the trends and correlates of occupational and leisure sitting time of full-time employed Australian adults. Approximately 51% of the respondents reported ≥4 hours of occupational sitting on a usual workday, 40% reported ≥4 hours of leisure sitting, and 55% reported ≥7 hours of combined occupational and leisure sitting. There were no clear trends over time. The associations with gender, age and leisure-time physical activity were reversed in direction for occupational sitting time and leisure sitting time.

Across all three surveys, the full-time employed respondents reported a mean self-reported occupational sitting time of approximately 4 hours/workday, a mean leisure sitting time of 3.5 hours/workday and a mean combined occupational and leisure sitting time of 7 hours/workday. This is worrisome, as seven to eight hours/day of total sitting time has been associated with increased risk of all-cause mortality in adults.[3] Furthermore, as the Australian Health Survey did not assess all domains of sitting time (such as transport-related sitting), it is likely that the current data underestimate the actual total sedentary time of this population.

The current study only included full-time employed respondents, only studied two domains of sedentary behaviour, and only focused on workdays. Therefore, the results are representative of the full-time employed Australian workforce only, and it is difficult to compare the results to previous studies involving general populations and which measured daily total sitting time. These earlier studies reported substantially lower levels of sitting time than in the current study, both in Australia and internationally. In the International Prevalence Study, for example, the median reported total sitting time was no higher than 5 hours/day, and 27% of the Australian respondents reported to sit >6 hours/day.[20] Moreover, 25% of Australian adults reported sitting >8 hours/day in the 45 and Up Study,[21] while 17% of Australian older women reported sitting >8 hours/day in the Australian Longitudinal Study on Women’s Health.[22] Finally, a European study reported that 18.5% of European adults reported sitting ≥ 7.5 hours/day, ranging from 9 percent in Spain to 32 percent in the Netherlands.[23] These differences with previous studies might indicate that levels of self-reported sitting time could be higher in full-time employed adults than in the adult population as a whole, when other domains are considered.

There was no clear trend across time for occupational sitting time, leisure sitting time, or the combination on workdays. Leisure time sitting seemed to have decreased between 2007/08 and 2011/12, but to have increased again in 2014/15. This was also reflected in the combined occupational and leisure sitting time numbers. For occupational sitting time, no changes were observed. It should be noted, however, that the proportion of full-time employed Australian adults, and thus the absolute population levels of sitting time, might have changed over time. Previous studies have reported opposing findings with regards to trends in sedentary time,[8–12] which demonstrates the need for continuous surveillance efforts of population levels of sedentary behaviour.

All potential socio-demographic and health-related correlates that were included in the analyses were associated with occupational sitting time. High household income and high educational level showed the strongest associations with ≥4 hours/day occupational sitting time. This is in line with previous research (in the general population, focused on total sitting time)[24, 25] and is likely explained by desk-based occupations and the associated high amounts of occupational sitting. Although less pronounced, all potential correlates were also associated with leisure sitting time, except for educational level. This might indicate that educational level does not influence leisure sitting time on a workday, at least not for full-time employed adults.

The directions of the associations with gender, age and leisure-time physical activity were reversed for occupational sitting and leisure sitting. Women, older people and people with sufficient levels of physical activity were more likely to accumulate ≥4 hours/day of occupational sitting; while men, younger people and inactive people were more likely to accumulate ≥4 hours/day of leisure sitting time.

To the authors’ knowledge, these differences in correlates of different sitting domains have not been studied before. The opposing results regarding gender might indicate that women more often have sedentary occupations than men, but sit less during leisure time. The finding that younger (15–34 years old) respondents had lower levels of occupational sitting might be influenced by a proportion of this group being employed from a young age, possibly in more physically active occupations. The negative association between leisure-time physical activity and leisure sitting time might, at least partly, be explained by time substitution; more leisure time spent on sedentary behaviours will leave less time to spend on physical activities.

These differences in correlates of different sitting domains might explain some of the inconsistent results that have been reported in the literature.[24, 25] Moreover, they emphasize the importance of assessing the different domains of sedentary behaviour in research and surveillance in order to gain a complete picture of the behaviour as well as the underlying determinants, and to subsequently address these different sedentary behaviour domains separately in interventions and policy. Future research should also consider the influence of new technologies (e.g. connectivity) on the distinction between sedentary domains (e.g. a change away from traditional working hours), and think about meaningful ways to measure new forms of sedentary behaviour and across domains.

### Strengths and limitations

The strengths of this study include the large population-representative samples of the Australian Health Surveys, and the repeated approach using consistent measures across the three surveys.

A limitation of this study is the use of self-report to assess sedentary behaviour. Self-report measures have limited validity due to issues with recall and social desirability.[26] Moreover, as the health risks of prolonged sitting have received quite some attention in Australia in recent years, this could have led to stronger social-desirability bias and consequently more under-reporting of sitting time in the more recent surveys. This could have influenced the trend analyses, possibly concealing changes in sitting time. Therefore, surveillance using a combination of objective measures such as accelerometers/ inclinometers that are not subject to social-desirability bias, and subjective measures to obtain contextual information would be preferable, even though applying these on such a large scale is challenging.

In addition, the Australian Health Surveys only assessed occupational and leisure sitting time, ignoring other domains of sedentary behaviour such as transport-related sitting (e.g. driving a car). Even though occupational and leisure-time sitting probably constitute the majority of total sitting time, especially in full-time employed adults, transport-related sitting time is an important source of sitting time for Australians.[10] Therefore, it is likely that the actual population levels of sedentary time in full-time employed Australian adults are higher than the levels reported in this study. Furthermore, as the Australian Health Surveys only asked about sitting time on a usual workday, it does not provide the opportunity to assess sedentary behaviours during non-work days and weekends, even though sedentary behaviours may be different on these days.[27]

Finally, even though we were able to describe trends across the three surveys, the number of surveys as well as the time intervals between the surveys was small. Therefore, continuous monitoring of these behaviours is essential to gain a better picture of the changes in the population levels.

## Conclusions

Over half of the full-time employed Australian adults reported levels of sitting time associated with increased risk for all-cause mortality in Australian Health Surveys conducted between 2007 and 2015. As transport-related sitting was not taken into account, these data probably underestimate the total sedentary time of this population. There were no clear trends over time for occupational sitting, leisure sitting, or the combination of occupational and leisure sitting time on workdays. Differences in correlates of occupational sitting time and leisure sitting time demonstrate the need to separately assess and target different sedentary behaviour domains in both research and policy.
